# Canonical template tracking: Measuring the activation state of specific neural representations

**DOI:** 10.3389/fnimg.2022.974927

**Published:** 2023-01-09

**Authors:** Ana F. Palenciano, Mehdi Senoussi, Silvia Formica, Carlos González-García

**Affiliations:** ^1^Mind, Brain, and Behavior Research Center, University of Granada, Granada, Spain; ^2^CLLE Lab, CNRS UMR 5263, University of Toulouse, Toulouse, France; ^3^Department of Experimental Psychology, Ghent University, Ghent, Belgium; ^4^Department of Psychology, Berlin School of Mind and Brain, Humboldt Universität zu Berlin, Berlin, Germany

**Keywords:** multivariate analyses, neural representation, canonical template, fMRI, EEG

## Abstract

Multivariate analyses of neural data have become increasingly influential in cognitive neuroscience since they allow to address questions about the representational signatures of neurocognitive phenomena. Here, we describe Canonical Template Tracking: a multivariate approach that employs independent localizer tasks to assess the activation state of specific representations during the execution of cognitive paradigms. We illustrate the benefits of this methodology in characterizing the particular content and format of task-induced representations, comparing it with standard (cross-)decoding and representational similarity analyses. Then, we discuss relevant design decisions for experiments using this analysis approach, focusing on the nature of the localizer tasks from which the canonical templates are derived. We further provide a step-by-step tutorial of this method, stressing the relevant analysis choices for functional magnetic resonance imaging and magneto/electroencephalography data. Importantly, we point out the potential pitfalls linked to canonical template tracking implementation and interpretation of the results, together with recommendations to mitigate them. To conclude, we provide some examples from previous literature that highlight the potential of this analysis to address relevant theoretical questions in cognitive neuroscience.

## 1. Introduction

Since the early 2000s, multivariate analyses of brain data have become a powerful tool for cognitive neuroscience (Norman et al., 2006; Naselaris et al., 2011; Haxby et al., 2014; Stokes et al., 2015; Cichy and Teng, 2017). Before the introduction of these techniques, the conventional approach in neuroimaging research (also known as the *activation-based framework*; Kriegeskorte et al., 2006; Mur et al., 2009) employed spatially aggregated activations means to describe the neural correlates of major *cognitive processes*. Hence, psychological functions could be identified with increases in functional Magnetic Resonance Imaging (fMRI) hemodynamic signal across macro-scale regions or increases in mean voltage amplitude registered with magneto/electroencephalography (M/EEG). In sharp contrast, the multivariate (or *information-based)* approach highlights that spatially distributed activity patterns contain—or represent (but see Carlson et al., 2018; Ritchie et al., 2020)—*information* used and manipulated by cognitive processes (Haynes and Rees, 2006; Mur et al., 2009). These techniques, grouped under the term **Multivariate Pattern Analyses** (**MVPA**, see Table 1 for a glossary of relevant terms), instead of averaging the signal, combine the activity registered across spatial units in a meaningful fashion to uncover neural patterns. Both the activation- and the information-based perspectives are not exclusive, but complementary (Hebart and Baker, 2018). Nonetheless, the latter has significantly influenced the research questions being addressed with neuroimaging and contributes to more mechanistic explanations of brain function. Therefore, the methodological advances in this field have grown exponentially over the last years. Here, we aim to extend these efforts, focusing on a specific multivariate technique, **Canonical template tracking (CTT)**, which seeks to identify the presence and strength of *specific representations* during the completion of cognitive tasks. The current work is motivated, on one hand, by the theoretical relevance of the CTT framework, and on the other, by the lack of clear guidelines on this technique in previous literature. In what follows, we first describe CTT in the context of more common multivariate analyses, to later provide a step-by-step tutorial on its implementation.

**Table 1:** Glossary with definitions of the main analysis labels used in the manuscript.

**Concept**	**Definition**
Multivariate pattern analysis (MVPA)	Term conveying all the analytical approaches that simultaneously treat multiple brain activity measurements. In most cases, these measurements are distributed across spatial units (i.e., voxels, channels, sensors…).
Decoding	Generally, decoding entails using brain data to predict experimental conditions, in opposition to encoding, which entails using experimental conditions to predict brain data (as in univariate General Linear Model estimation). In this work, we use the term decoding to refer more specifically to its most popular implementation, in which machine-learning classifiers are trained and tested on brain activity patterns. It informs about how discriminable two (or more) experimental conditions' activity patterns are.
Cross-decoding	Implementation of decoding analysis in which the machine-learning classifiers are trained and tested on datasets obtained in different experimental conditions (i.e., training the classifier with data from task A and testing it with data from task B). It informs about how generalizable the encoding space is among conditions.
Representational similarity analysis (RSA)	MVPA technique that computes (dis)similarity measurements among activity patterns from all pairwise combinations of experimental conditions (or stimuli, trials, etc.). It can be performed both in a model-based (using explicit similarity predictions) or an exploratory fashion. It informs about the overall geometry or structure of representational spaces.
Canonical template tracking (CTT)	MVPA technique that combines either decoding or RSA computations with independent localizer task(s). It is based on the estimation of canonical templates from the localizer(s) data, and its comparison with the main paradigm activity patterns using either machine-learning classifiers or similarity measurements. It informs about the activation of representations with specific content and/or under specific format.
Functional localizer	Tasks designed to isolate brain region(s) engaged by specific cognitive processes. This methodology, used in the univariate analysis tradition, has enabled restricting the analyses of independent datasets (i.e., from a main experimental paradigm) to a series of functional regions of interest, defined individually for each participant.

**Figure 1:**
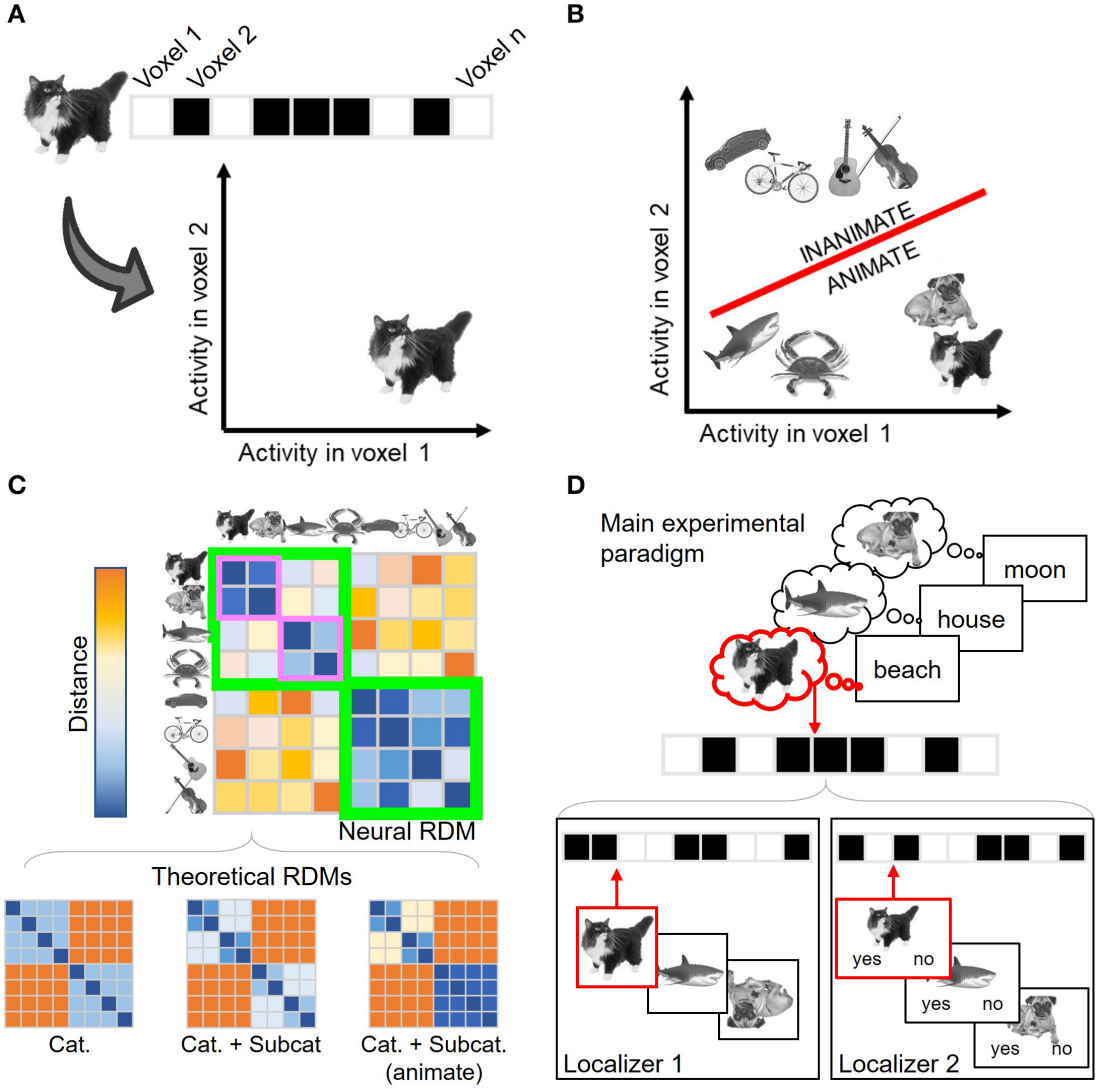
Overview of the different multivariate analyses. **(A)** Neural representations can be understood as points in an *n*-dimensional space, where n corresponds to the number of spatial units considered. A two-dimensional (i.e., two voxels) space is shown here for visualization purposes. Each condition (in this example, each stimulus) is defined by its associated activation across the spatial units. **(B)** Decoding analysis seeks to assess how differentiable are the conditions' representations in this space. To do so, a boundary (shown in red) that separates two (or more) conditions is first estimated and later tested with independent, unlabeled activity patterns. In this example, the boundary classifies between two category conditions: animate and inanimate stimuli. **(C)** Representational Similarity Analysis (RSA) describes the geometry of the space computing the similarity structure across conditions. In the example, the estimated similarity matrix shows that the representations are organized not only by the stimulus category (animate and inanimate stimuli, lower and upper quadrants shown in green) but also by the animate stimulus subcategory (mammals and non-mammal animals, shown in purple). RSA also enables characterizing the estimated encoding space using theoretical models. In this case, the third model (which predicts a broader animate/inanimate distinction together with further animate subcategory differentiation) will better predict brain data. **(D)** Canonical Template Tracking (CTT) estimates specific canonical neural representations using localizer tasks (lower panels) and then assesses their activation level during the main paradigm (upper panel). In the example, the paradigm is a memory retrieval task in which the participants retrieve the images that were associated with each word in a previous learning phase. The stimuli sensory templates (Localizer 1) are estimated with a task where upside-down images need to be detected, while semantic templates (Localizer 2) are estimated with a task in which the stimuli are categorized as mammals or non-mammals. Those templates are then compared against the activity patterns associated with the items retrieved during the main paradigm. Critically, the activation strength from the retrieved image template (in the example, the cat, shown in red) can be compared against the non-retrieved ones (e.g., the dog templates), which can act as baseline condition. Cat., Category; Subcat., Subcategory. Stimulus images were retrieved from the stimulus database employed in González-García et al. (2021), CCBY 4.0.

All MVPA approaches depart from the assumption that information is encoded in the distributed patterns of activation across spatial units (fMRI voxels, M/EEG channels, etc.). In this context, neural patterns (which may coincide with our experimental conditions, stimuli, trials, etc.) are considered points in a high-dimensional representational space (Haynes and Rees, 2006), whose axes are defined by the activity in the spatial units considered (Figure 1A). Each multivariate technique explores the resulting representational space differently, providing a distinct insight into neural encoding. **Decoding analyses** (Norman et al., 2006; Haxby et al., 2014), the first popularized multivariate approach (Haynes and Rees, 2005; Kamitani and Tong, 2005; Haxby, 2012), employ machine-learning classifiers to address whether two or more conditions' representations can be disentangled in this space (Figure 1B). These classifiers are algorithms trained to establish a boundary that separates multidimensional neural activity patterns elicited by different experimental conditions. The classifiers are later tested against new data, i.e., not used in the training phase, to estimate how accurately they can predict the experimental condition of unlabeled activity patterns. Significant above-chance decoding performance informs us of the *content* of brain representations. For instance, if a classifier can successfully discriminate between activity patterns elicited by two different stimulus categories, it provides evidence in favor of the neural representation of category information. Under well-controlled experimental designs, the accuracy values can also reflect the strength of such representations, i.e., how large are the differences between the conditions' neural patterns (although see Hebart and Baker, 2018).

Nonetheless, a downside of decoding analyses is that the classifiers remain agnostic regarding the structure of the representations leading to a significant classification. Hence, this approach reduces the comparison between conditions to an index of how discriminable they are (i.e., decoding accuracy) but without specifying how the neural patterns relate to each other. This is exemplified by the fact that several different representational spaces could still provide similar classifier performance (Kriegeskorte and Kievit, 2013). This motivated the formalization of **Representational Similarity Analysis** (**RSA**; Kriegeskorte et al., 2008a; Kriegeskorte and Kievit, 2013), another popular MVPA technique that aims to estimate and describe the *structure* (or *geometry*) of representational spaces. This technique computes how similar (or, alternatively, how dissimilar or distant) the patterns of pairs of conditions are in the activity space. These similarity metrics are then conveyed in neural representational similarity matrices (Figure 1C), which capture the dimensions organizing the representational space. Although these matrices can be directly explored using data visualization techniques (Kriegeskorte et al., 2008a), they can also be compared against theoretical models, which are flexibly generated using predictions from theories, computational models, behavioral performance, deep neural networks, etc. This comparison is carried out by computing additional second-order similarity measurements between neural and theoretical similarity matrices (Kriegeskorte et al., 2008a; Nili et al., 2014), which provide insight into which and to what extent theoretical models explain variance in neural data.

Aiming to extend these two general approaches, here we describe a third MVPA implementation that combines aspects from both decoding and RSA and embeds them in the **functional localizer** logic. For sake of consistency with previous studies (Wimber et al., 2015; González-García et al., 2021), we refer to this technique as CTT. The employment of localizers (Saxe et al., 2006) has been a common practice in the activation-based perspective to isolate brain regions involved in cognitive processes (for instance, face localizers aiming to identify subject-specific face fusiform area; Kanwisher et al., 1997). In a similar vein, CTT employs localizer tasks from a multivariate perspective, where each localizer task is meant to elicit specific neural representations. From the localizers' data, a series of canonical neural templates are estimated to capture the standard neural representations of particular types of information (Figure 1D, lower panel). Then, CTT detects whether and the extent to which these templates are present during cognitive tasks. To do so, the canonical templates are compared against the activity patterns evoked by a main experimental paradigm (Figure 1D, upper panel). For that comparison, CTT incorporates analytical tools provided by decoding analyses and RSA. One possibility is to train machine-learning algorithms with the localizers' activity patterns and testing them against the main task data, to assess the presence or absence of encoded information (e.g., Senoussi et al., 2016; Kok et al., 2017; Liu et al., 2019). Moreover, similarity measures derived from RSA (Walther et al., 2016) can also be computed between the localizers' and the main paradigm's activity patterns to inform about the activation strength of the individual templates' representations (Wimber et al., 2015; González-García et al., 2021).

To better understand the CTT procedure, we describe the experiment carried out by Wimber et al. (2015), which illustrates the advantages of including localizer tasks within the MVPA logic. In their study, the authors aimed to interrogate the phenomenon of retrieval-induced forgetting, i.e., how retrieving certain information from long-term storage leads to the adaptive forgetting of competing memories. To do so, they first trained their participants to learn a series of associations among words and pairs of images. Then, inside the fMRI scanner, the participants saw the words and were instructed to remember only one of the associated images. That induced a poorer recall of the second image, which acted as a competitor in this task. To test previous theoretical accounts of retrieval-induced forgetting, the main goal of the study was to estimate the activation strength of both retrieved and forgotten image representations while the associated word was on the screen. To do so, the authors conducted a localizer task to estimate the activity patterns (i.e., the canonical templates) linked to all the images used in the experiment, including both target and competing stimuli. Then, they computed the similarity (using correlations) between templates of both experimental conditions and the activity patterns maintained during the retrieval-induced forgetting phase. Their findings evidenced a greater reinstatement of target image representations, together with a greater de-activation of competing image templates.

This study highlights the main benefit of incorporating localizer tasks with the CTT logic: its ***specificity***. Like other MVPA approaches, CTT taps into the *content* of brain representations. Nonetheless, while regular decoding does so more generally—by identifying whether activity patterns are systematically different among experimental conditions—, CTT identifies the presence of neural representations linked to more constrained cognitive constructs. In the case of the experiment by Wimber et al. (2015), it allowed identifying the representational signatures of individual stimuli to assess and compare their activation strength. In this regard, although decoding has proven to be a powerful technique to capture the broader classes that structure perceptual information (e.g., Haxby et al., 2001; Reddy and Kanwisher, 2007), this level of specification is more difficult to achieve. The most used performance metric—i.e., the classification accuracy—would not inform on the status of individual representations, but rather, about the overall distance among categories. In contrast, RSA, especially in combination with condition-rich designs (i.e., using a wide number of conditions, sparsely sampled), can capture fine-grained encoding structures computing dissimilarities among pairs of specific stimuli or experimental material (e.g., Connolly et al., 2012). However, the standard implementation of this approach leads to the estimation of an overall encoding structure, computed from the whole dataset (or chunks of it, such as independent scanning sessions). Hence, the extraction of stimuli (or condition)-wise representational status is hindered with this approach (although see Nili et al., 2020). In contrast, and as it is illustrated in the above-mentioned study by Wimber et al. (2015), the employment of localizer tasks and similarity measurements can facilitate access to individual representations' activation strength.

Furthermore, the increased specificity enabled by CTT allows us to explore not only which content is encoded, but also in which ***format***. The representational format, which constitutes a key dimension for several theoretical frameworks (e.g., Jeannerod, 2001; Barsalou, 2008), refers to the type of code (e.g., perceptual, motor, abstract, etc.) in which the information is maintained during different cognitive operations. This attribute is more difficult to identify with other multivariate analyses since showing that certain information is successfully classified (with decoding) or follows a particular organization (with RSA) does not necessarily inform about the underlying representational format. For instance, two conditions (e.g., animate and inanimate targets) could be equally segregated irrespectively of their format (e.g., perceptual or semantic). An equivalent situation could take place regarding the similarity structure found across conditions (e.g., under both representational formats, higher distances could be expected among stimuli from different categories than among stimuli from the same category). In contrast, imposing different task demands across independent localizers can help in encapsulating representational formats in canonical templates (González-García et al., 2021). Moreover, using overlapping material under different task demands across localizer tasks can further help in disentangling between encoding content and format (see González-García et al., 2021). This is exemplified in Figure 1D, where the two localizers illustrated in the lower panel aim to target the representations of overlapping stimuli (in the example: cat, octopus, dog) in distinct encoding formats (sensory and semantic templates) by manipulating the task demands. Hence, thanks to the thoughtful design of localizer tasks, CTT can be a powerful technique to assess the activation of *specific* content under a *specific* format.

CTT can be further advantageous for more particular research questions. First, it can go beyond traditional decoding in describing representational content when the ***ground truth remains uncertain*** for the researcher. There are cognitive paradigms in which there is no one-to-one correspondence between brain data and experimental conditions. For instance, the targeted cognitive process may entail the simultaneous coding of several representations, while the likelihood of each information activation strength may remain unknown. That was the case in the study by Wimber et al. (2015), where a single activity pattern could reflect both enhanced and inhibited information encoding. In such circumstances, each activity pattern cannot be described by a unique and true label. Therefore, the traditional decoding procedure could not be carried out: the classifiers could not be trained nor tested in the absence of proper labels. In contrast, CTT would bypass that issue, since it allows the estimation of the full spectrum of candidate representations (i.e., as many as we include in our localizer), and then directly computes which ones are being instantiated, and to what extent (as in Wimber et al., 2015). Related to this topic, the cognitive process of interest may activate one out of many representations, although its identity cannot be experimentally manipulated. For instance, in bistable perception, the content perceived by the participant fluctuates spontaneously, and unless a response is required, the researcher is agnostic to that information (Sterzer et al., 2009). A more extreme scenario takes place when the content of spontaneous activity patterns is studied (Liu et al., 2022), such as in unconstrained imagination or dreaming (e.g., Horikawa et al., 2013). Even when the participants' report can inform about the content linked to different time points of the brain data collected, the potential diversity of engaged representations, together with the lack of control of the experimental context, would hinder the implementation of standard decoding protocols. In this regard, the employment of localizers has been recently identified as a suitable method for research in more naturalistic and spontaneous brain functions (Liu et al., 2022).

Second, CTT can also be a powerful technique to interrogate representational geometry in situations where ***theoretically driven predictions are difficult to establish***. While RSA has been successfully used to estimate the structure of information encoding from neuroimaging data, one of the most frequent implementations, model-based RSA, depends on the availability of theoretical predictions to formally describe representational spaces. Although there is also a more data-driven version of RSA which directly explores the encoding geometry, the model-based modality would be hindered when we are agnostic regarding representational structure. While CTT substantially overlaps with RSA (i.e., by using RSA-derived similarity measurements to compare multiple canonical templates), it can be particularly advantageous in such circumstances. Thanks to the acquisition of independent localizer blocks, where the task demands are explicitly manipulated, this framework enables the direct estimation of empirically-obtained representational spaces emphasizing different cognitive dimensions. Moreover, model-based RSA could be also difficult to apply in circumstances where we aim to address the impact on representational geometry of latent variables that were not explicitly manipulated in the experimental design. The work by González-García et al. (2021) illustrates this situation. In this study, the authors aimed to describe the encoding format for novel stimulus-response associations, disentangling between procedural and declarative mappings representations. Importantly, adding the encoding format as an explicit factor in the study's design could distort the cognitive processes involved in the main paradigm, and hence, not help address the research question. As a result, even when the two encoding formats' representational geometry could be predicted, the ***theoretical modes shared the same similarity structure***. Specifically, both theoretical RDMs predicted higher similarity between same-conditions trials (e.g., between associations involving the same stimulus category and response conditions) than between different-conditions trials (e.g., between associations involving the different stimulus category or response conditions). Hence, the similarity structure could not disentangle between procedural and declarative formats, even when the underlying multivariate activity patterns could differ. Instead, CTT allowed inducing the two encoding formats in separate localizers, and then track the localizers' activity patterns (or canonical templates) instead of the similarity structure. Nonetheless, it is also important to stress that more sophisticated experimental designs, such as condition-rich designs (Kriegeskorte et al., 2008b; Connolly et al., 2012; Nastase et al., 2017), can enable the orthogonalization of latent dimensions (e.g., content and format) and provide a solution for this issue. However, while these designs can be easily incorporated in domains such as visual perception (e.g., Kriegeskorte et al., 2008b), their use in other research contexts can be more challenging, as for higher-order cognitive processes (Freund et al., 2021). For all the above-described circumstances, including localizer tasks designed to convey the alternative representational geometries could be an interesting option. Overall, despite the methodological overlap between RSA and CTT, the latter further allows the estimation of representational spaces to characterize neural encoding, as well as the direct comparison with the templates' activity patterns.

Finally, another core aspect of CTT is the ***generalizability*** of its results (Varoquaux and Poldrack, 2019), thanks to the comparison of activity patterns across independent tasks. In this sense, CTT is highly similar to **cross-decoding** (Kaplan et al., 2015), a variant of decoding analysis that assesses whether an algorithm trained in a given task successfully classifies activity patterns in a different one, evidencing a common neural code. Both CTT and cross-decoding aim to extrapolate representational spaces across contexts to better characterize them. However, cross-decoding is usually employed in single-paradigm studies, comparing activity patterns from different experimental conditions. As a result, the comparison is performed among a limited set of tasks: in most cases, between two contexts. Including localizer tasks within this logic can be beneficial for two reasons. First, it can facilitate the estimation of more constrained representations of interest, and second, using localizers provides a more flexible framework in which the contribution of several templates (either from the same or from different localizer tasks) can be considered simultaneously. Additionally, CTT will not only inform on whether the representational space generalizes (as above chance cross-decoding performance does). By computing similarity measurements, it can provide further evidence for the generalization of each individual template included in the analyses. Hence, while the two techniques are tightly related, CTT may overcome standard cross-decoding in some research fields.

Hence, while decoding and RSA are both powerful approaches to characterize the content and geometry of neural representations, CTT should be also considered as a complementary approach that extends the theoretical scope of previously documented multivariate brain data analyses. However, despite approaches akin to CTT have been present in the literature since the early beginnings of the information-based approach (Polyn et al., 2005), no work to date attempted to provide a comprehensive methodological description of this technique. We believe that providing clear guidelines on how to implement this analysis is still required to contribute to better practices with this method (as has happened with other facets of MVPA; for instance, see Gorgolewski et al., 2016). Aiming to fill that gap in the literature, here we provide a step-by-step tutorial on CTT. We will first focus on important design issues regarding the nature of the localizer tasks, to later expose the relevant analytical decision that should be made during the process. In doing so, we cover issues that affect spatially resolved (fMRI) and time-resolved (M/EEG) datasets. We further provide a series of scripts, compatible with one of the most popular multivariate analysis software, The Decoding Toolbox (TDT; Hebart et al., 2014), to implement this analysis. Finally, we conclude by illustrating some examples where the CTT approach has helped test cognitive neuroscience hypotheses and highlighting research fields where CTT may be particularly relevant in future research.

## 2. Design considerations

When designing CTT experiments, a series of key decisions will concern the nature of the localizer task(s). Localizers should evoke the representations of interest, regarding both content and format. Imagine, for instance, that we aim to assess the presence of sensory stimulus information during a given cognitive paradigm. In that case, the localizer should trigger these representations by using a task that somehow entails the visual perception (*format*) of the stimuli of interest (*content*). A wide diversity of localizer tasks has been used in previous research framed within CTT, mostly inspired by the most popular functional or univariate version of this approach. For instance, to capture perceptual or sensory representations (mostly in the visual domain), the localizers employed have entailed passive stimuli viewing (e.g., Liu et al., 2019), discrimination tasks, focused on either physical (e.g., Wimmer et al., 2020) or conceptual (e.g., Treder et al., 2021) attributes of the stimuli, or n-back tasks (e.g., Wimber et al., 2015). Similarly, sensorimotor representations have been isolated with localizers where participants execute responses with the effectors of interest (e.g., Henderson et al., 2022). More abstract attentional or task-level representations have been also targeted using complex localizers which incorporate task goals as a relevant dimension (e.g., Collins et al., 2014; González-García et al., 2021). In clinical contexts, approaches similar to CTT have been followed to isolate the neural fingerprints of pain and assess their predictive value (Wager et al., 2013). Overall, this diversity illustrates the flexibility inherent to this framework, while also stressing the lack of consensus on how to best capture canonical representations across different domains. Aiming to provide some guidance in the design of future CTT studies, in what follows we will address three main sets of decisions, involving (1) the localizer task demands; (2) the localizer (experimental and control) conditions; and (3) the overall temporal structure of the experiment.

### 2.1. Localizer task demands

The task demands imposed in the localizer block(s) will generally determine the encoding format of the canonical templates. A crucial step to define them will be to carefully decompose the main experimental paradigm and the tentative localizer tasks into the cognitive processes that they engage, to ensure that the localizer(s) overlap at the process of interest (but also see Friston et al., 1996). Nonetheless, while the triggered cognitive process can be very diverse among studies, we could still choose localizers that share more or fewer task components with the main paradigm. On one side of the spectrum, using localizer(s) very similar to the main paradigm (for instance, using similar perceptual features, motor responses, and/or cognitive demands) would increase the likelihood of finding shared representations. However, that is achieved at the cost of losing the specificity of CTT (i.e., constraining the activity patterns), leading to more general results induced by other processes common among tasks but outside the focus of the research question. For instance, if we aim to track perceptual representations, but similar motor responses are required in both the main paradigm and in the localizer (as it happens, for instance, when the localizer requires a response in every trial), finding overlapping activity patterns could reflect motor preparation or execution, or stimulus-response binding. Similarly, in the motor domain, keeping similar visual displays between a motor response localizer and the main paradigm could also contaminate the generalization. On the other side of the spectrum, however, the localizer(s) can be very different from the main paradigm, which would maximize the generalizability of the findings (Varoquaux and Poldrack, 2019). Nonetheless, it can also invoke additional processes that could distort the canonical templates, imposing a much more stringent test. For example, using an n-back task (as in Wimber et al., 2015) with infrequent repetitions after n trials will ensure a high percentage of observations where no responses are executed. That will allow the isolation of perceptual representations avoiding the above-mentioned issue with unwanted motor confounds. In turn, it will add additional working memory demands (maintaining the past n-back item), which may contaminate the estimated templates and impoverish the generalization. In consequence, the trade-off between sensitivity and specificity induced by the similarity between the main paradigm and localizers should be thoughtfully considered during the design stage. Adjusting this trade-off would be particularly important if multiple localizers are included in the design, ensuring that all of them are equivalent regarding their similarity to the main paradigm. In a similar vein, behavioral performance and task difficulty should be kept as constant as possible across localizer blocks.

### 2.2. Localizer conditions

The second set of decisions will involve clearly identifying the different experimental conditions that will translate into individual canonical templates, as well as the conditions that will be as baseline or control.

The conditions of interest will define the content of the templates, and as such, should cover the full spectrum of representations that we aim to track during the main paradigm. Hence, the identity of these conditions (i.e., the perceptual, motor, or cognitive information) should be clearly defined. Regarding that, a key aspect to consider is how fine or coarse-grained these representations are. We may aim to capture canonical templates of particular exemplars (for instance, of a specific face identity or a left index flexion movement) or broader categories (for instance, female faces or left-hand responses). Connecting with the previous section, and especially in localizers that include active demands, it is important to consider the alignment between the experimental conditions and the dimensions that are behaviorally relevant to the participants. The tasks employed should emphasize the features that differentiate the target templates that we aim to estimate. For instance, using a localizer with a discrimination task where the relevant behavioral dimension is stimulus category (e.g., categorizing between animate and inanimate stimulus) would benefit the estimation of coarse-grained, categorical neural templates, while it could not be optimal for the estimation of finer-grained stimulus representations. Instead, the latter could be more accessible using an alternative task requiring the processing of exemplar-wise attributes. Overall, the likelihood of finding an activation of these templates during the main paradigm will depend on whether the demands align (following the previous example, it will depend on whether the stimulus category is a relevant dimension both during the main task and the localizer).

Critically, the CTT implementation will also depend on defining control conditions within each localizer task, which will be used as baseline to assess the effect of each canonical template. Even after carefully designing the localizer demands, finding a systematic relationship between a particular canonical template and the main paradigm data could reflect the activation of the template content, but also more general task processing (see González-García et al., 2021). For instance, if we estimate canonical templates for different animate and inanimate stimuli and just assess the reinstatement of each of them during another task, we may capture the encoding of individual stimuli, but also more general perceptual processing mechanisms. To rule out these unwanted effects and ensure the content specificity of the CTT results, the templates are compared against proper control conditions. Hence, relative (instead of absolute) activation indexes are interpreted (see Section 3.5). Moreover, the use of control conditions is also important for certain measurements included in the CTT framework (see Section 3.3) that cannot be interpreted in an absolute fashion and require a control condition to assess their relative change. Depending on the localizer, different baseline conditions can be designed. A parsimonious approach consists of using trial-irrelevant conditions templates as baseline. For instance, if we estimate templates for animate and inanimate stimuli, we can compute the difference between their activation in trials from the main paradigm where only one template condition (e.g., animate) is relevant, employing the other condition (e.g., inanimate) as baseline (see the provided scripts for a demonstration of this approach). Nonetheless, more sophisticated design options can also be followed, such as using a neutral condition (for instance, presenting stimuli that did not appear in the main paradigm) that enables addressing both increments and decrements in the template's activation strength (as in Wimber et al., 2015). Overall, these relative measurements will provide stringent evidence in favor of the presence of an individual template.

### 2.3. Temporal structure

The temporal structure in which the main paradigm and localizer block(s) are presented should also be considered. The simplest option will be to group all the localizer tasks together and to present either the main paradigm first with the localizer block(s) at the end (e.g., González-García et al., 2021) or the other way around (e.g., Liu et al., 2019). This option can be optimal if exposure to either the main paradigm or the localizers can bias performance somehow, or when the content of the localizers is defined a posteriori, based on the participants' execution. Moreover, if several localizer tasks are employed, localizer order should also be controlled in our design. In this regard, we recommend counterbalancing the sequence across participants, although the efficiency of this approach will depend on the inference performed (Todd et al., 2013). Finally, while locating the localizer(s) at the beginning or the end of the experimental session is straightforward to implement, it can be subject to contaminating effects that evolve over time, such as participants' fatigue or practice, or low-frequency noise from the neuroimaging technique employed. To prevent that, the main paradigm blocks can be alternated with the localizer(s) during the experimental session (e.g., Peñalver et al., 2022). In that case, further control on task (localizer, main paradigm) order should be implemented.

Finally, slow drift artifacts can be also confounded with time-on-task. In this regard, fMRI enables splitting the localizers into independent scanning runs (as it has been done in experimental designs for decoding analyses; Misaki et al., 2010; Coutanche and Thompson-Schill, 2012), which can mitigate this artifact effect. That is not the case for techniques such as EEG, where intermixing localizer with main paradigm blocks could be a suitable option. Taking all that into account, we further stress the use of relative measurements during CTT to mitigate the impact of more general, confounding effects induced by block order, fatigue, or practice.

To summarize, four core principles should guide the design of CTT studies. First, to achieve an optimal balance between specificity and selectivity, our general recommendation is to use localizers identical to the main paradigm only regarding the aspects (perceptual, motor, or cognitive demands) key for the representational format of interest, while maintaining the rest of the localizer task as parsimonious as possible (i.e., avoiding additional, unwanted processes). Second, we recommend defining the templates' experimental conditions not only by means of the material provided to the participants (e.g., perceptual stimuli) but also focusing on the relevant behavioral dimensions in the task (i.e., which features will be processed during the localizers). Third, and critically, adequate baseline or control conditions should be considered during the study design to ensure the specificity of the CTT results. Fourth, the sequence in which the main paradigm and localizer(s) block(s) are presented should be defined considering, among other aspects, the targeted cognitive processes (i.e., whether or not it is affected by the localizers' presentation order) and the neuroimaging technique employed.

More particular design considerations regarding the main paradigm will depend on whether the CTT procedure is implemented as an extension of RSA or decoding (see Section 3.3). In the former case, condition-rich designs are more suitable (Kriegeskorte et al., 2008a), enabling the investigation of finer-grained representations. Conversely, if classifiers are used for template tracking, the preferred designs involve few, broader conditions, maximizing the number of observations per condition. However, the lack of previous methodological research complicates estimating an a priori minimum number of observations required by the localizers (neither its ratio against the main paradigm's number of observations). In this regard, we recommend extensive piloting of the localizer tasks, together with the identification of relevant sanity check or control analyses that can be performed on pilot data to address the reliability of the canonical templates (see Section 3.5). Finally, more general indications for MVPA also apply to the CTT framework, such as splitting the main paradigm into separate (and equivalent) runs whenever cross-validated template tracking measurements are computed (Walther et al., 2016), or carefully controlling that all nuisance variables are balanced across conditions (Hebart and Baker, 2018).

## 3. Step-by-step tutorial on CTT implementation

**Figure 2:**
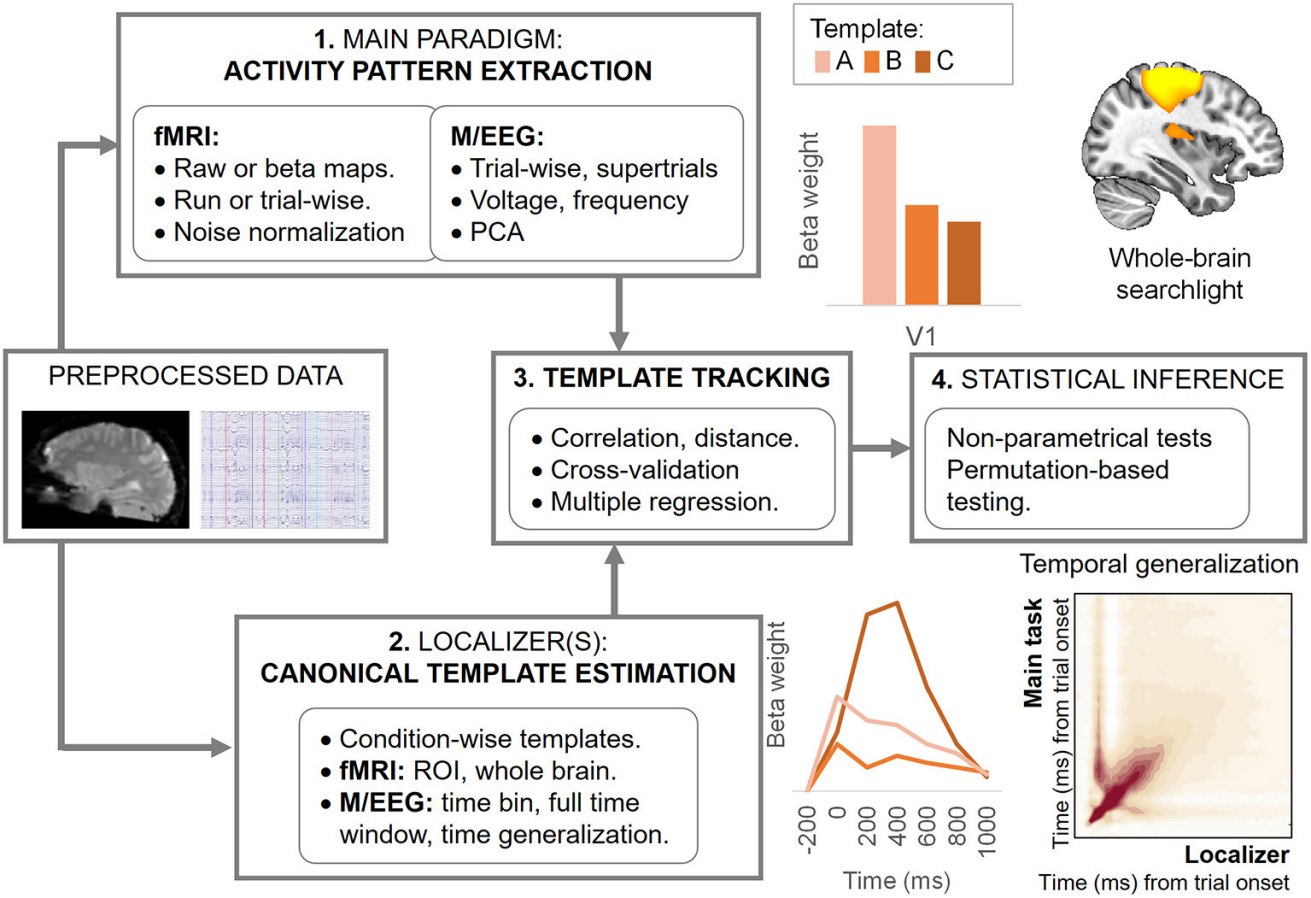
Analysis steps during canonical template tracking. The figure synthesizes the analysis protocol, from the data preprocessing to statistical inference. Insets figures, at the right, show different predicted outputs that could be obtained with CTT when applied to spatially resolved **(upper section)** and temporally resolved **(lower section)** techniques. The upper section shows examples from ROI **(left)** and searchlight-based **(right)** analyses. The lower section shows examples of the time course of different templates activation indexes **(left)** and a temporal generalization matrix **(right)**.

All CTT experiments provide at least two independent datasets per participant: one for the main experimental paradigm and another for the localizer. Before starting the CTT protocol, raw data will be preprocessed following the general recommendations for multivariate analyses. In the case of fMRI data, and according to the standards in the field, we recommend not normalizing nor smoothing the data before the analyses to avoid distortion in the activity patterns (Kriegeskorte et al., 2006; Mur et al., 2009, but see Op de Beeck, 2010; Gardumi et al., 2016). For time-series data (EEG, MEG), we refer to previous work assessing the impact of different preprocessing steps on distributed activity patterns (Grootswagers et al., 2017; van Driel et al., 2021). Nonetheless, the preprocessing pipeline falls outside the scope of the current work, and we will focus instead on the analysis and statistical inference, whose steps are summarized in Figure 2. Code to implement the analyses described in this tutorial is available in the following GitHub repository: https://github.com/AnaPalenciano/Canonical_Template_Tracking.

### 3.1. Extraction of the main paradigm activity patterns

The first step in CTT is common to other MVPA techniques and consists of the extraction of the activity patterns from the main paradigm data. This procedure generates a series of conditions-wise N-length vectors, where N corresponds to the number of spatial units (voxels, channels, etc.) considered, containing the activity level of each spatial unit. Currently, there are several approaches to extract these activity patterns. Although all of them are in principle valid for both space and time-resolved techniques, different methods have been used in the fMRI and the EEG/MEG literature (Mur et al., 2009; Pereira et al., 2009; Grootswagers et al., 2017).

In fMRI, the simplest approach consists of directly using the raw images and extracting the voxel-wise changes in Blood-Oxygen-Level-Dependent (BOLD) signal (Polyn et al., 2005; Pereira et al., 2009). However, the most frequent practice is to estimate a General Linear Model (GLM; Friston et al., 1994, 1995) which decomposes each voxel's signal variance according to the different task conditions and nuisance regressors, and to use the estimated beta weights to generate the activity patterns. Two main modalities of GLMs are currently used for multivariate pattern extraction. The most conventional collapses across trials within scanning runs and generates a single beta map (and thus, a single activity pattern) per experimental condition and run, while a more recent implementation, which further controls for model collinearity (Mumford et al., 2012, 2014; Arco et al., 2018), enables trial-by-trial activity pattern estimation. The decision between these two options will depend on the experimental design and the template tracking approach followed. The former estimates fewer, but more stable activity patterns and is a better fit for designs with fewer but highly sampled conditions. The latter increases considerably the number of generated activity patterns, which can be beneficial in condition-rich designs and for intensive classifier training and testing procedures. Critically, while run-wise activity patterns are more robust, they do not allow exploring more dynamic trial-by-trial changes in neural representations.

Additionally, two further noise correction methods have been proposed to optimize fMRI activity patterns (Misaki et al., 2010; Walther et al., 2016). Univariate noise normalization, which simply entails using the *t* values linked to each beta weight, controls for the variance of individual voxels separately. It has been shown to improve the discriminability of activity patterns (Misaki et al., 2010). More recently, multivariate noise normalization has been also suggested as a potential strategy to mitigate spatially distributed noise (Walther et al., 2016, but see Ritchie et al., 2021). This method is carried out using the covariance across voxels from the GLM estimation residuals, and its computation has been recently added to available software for multivariate analyses (Hebart et al., 2014).

In contrast with the fMRI literature, activity pattern estimation has been substantially less documented for temporally-resolved recording techniques such as EEG and MEG. In line with the tradition on univariate Event-Related Potentials (ERPs), the time-series data is segmented into trials, to then extract the trial-wise voltage amplitude or (time-)frequency power (e.g., Quentin et al., 2019; Senoussi et al., 2020) across channels, independently for each time point sampled. Nonetheless, it has been recommended to perform some sort of trial averaging (also referred to as *supertrials*), which significantly increases the signal-to-noise ratio (Isik et al., 2014; Grootswagers et al., 2017; Hebart et al., 2018; Senoussi et al., 2020). While there is not a clear consensus on previous literature regarding supertrial generation, averaging across reduced-size sets (as small as four trials) already provides a significant improvement in pattern discriminability (Grootswagers et al., 2017). Additionally, and to further attenuate noise, the activity patterns can be temporally smoothed (Larocque et al., 2012; Hebart et al., 2018; López-García et al., 2022) before being fed into the main analyses. The noise normalization techniques proposed for the fMRI field had been also extended to temporally-resolve datasets (Guggenmos et al., 2018).

Due to the lower spatial resolution of EEG and MEG, the most common practice is to generate the activity patterns using the totality of channels registered. Nonetheless, as the number of channels increases (especially in MEG recording), so does the dimensionality of the estimated representational space, which can have a detrimental impact on further analyses. This motivated the employment of dimensionality reduction techniques, mostly Principal Components Analysis (PCA), to generate activity patterns from less and more informative features, without imposing further anatomical assumptions (Hebart et al., 2018). Extracting the activity patterns from the components accounting for 99% of the data variability has been evidenced to significantly improve the signal-to-noise ratio (Grootswagers et al., 2017).

Finally, as happens with other modalities of multivariate analyses, CTT can also be performed in the frequency domain through time-frequency decomposition of single-trial M/EEG activity. This allows to obtain activity patterns (i.e., sensor or source-reconstructed topographies) for each frequency/frequency band of interest, for instance, beta/mu activity reflecting motor processes (e.g., Murphy et al., 2021).

### 3.2. Estimation of the canonical templates

The second step in CTT is to estimate the canonical neural templates from the localizer task(s). This procedure is similar to pattern extraction performed on the main experimental paradigm data. Nonetheless, the former is more flexible, allowing the researcher to choose whether multiple (trial-wise) or single (run-wise) conditions patterns were computed. In the case of canonical neural template estimation, the preferred option is to obtain more robust and stable single activity patterns. Thus, we recommend employing single beta estimates for each localizer condition in the case of fMRI data (potentially improved with univariate or multivariate noise correction) and condition-wise averaged multi-channel activity patterns in the case of M/EEG recordings (either in time, frequency or time-frequency domain). Nonetheless, if the tracking is performed using a decoding-based approach (see Section 3.3), this stage should generate localizer data suitable to train the machine-learning classifiers. Hence, instead of a single, robust canonical template, several exemplars of each template condition should be estimated from the localizer data (more similar to the main paradigm activity pattern extraction).

A decision of particular importance in this step is to select either where in the brain (in the case of fMRI data) or when in time (in the case of time series data) are the canonical templates estimated. In the spatial domain, the region(s)-of-interest (ROIs) strategy accommodates well in the CTT framework since it can constrain the canonical templates generation to functionally relevant regions. In this case, for each ROI, the templates are estimated using all the voxels assigned to that region (see Figure 2, left upper inset). Nonetheless, whole-brain analyses, carried out with searchlight procedures (Kriegeskorte et al., 2006), can be also implemented. To do so, a sphere (whose ratio, in voxels or millimeters, is predefined a priori) is iterated across all the locations in the brain to extract the canonical templates and perform the analysis. In each iteration, the output of the analysis is assigned to the voxel located on the sphere center (see Figure 2, left right inset). While deciding between these two options will depend on the study hypotheses, it is important to stress that the same voxel selection criteria should apply also to the main paradigm activity pattern extraction. While recent methodological advances in the field have enabled establishing relationships between different regions' activity patterns (Ito et al., 2017; Karimi-Rouzbahani et al., 2022), the CTT method proposed here is so far restricted to within-region comparisons. Further investigation is still needed to validate its application using data from different brain regions.

Regarding the temporal domain, the most frequent approach in multivariate testing has been to explore the full epoch of interest, extracting independent activity patterns at each time bin sampled. The same logic could be followed with CTT, dynamically estimating the canonical template at each individual time point (see Figure 2, left lower inset). In this regard, the CTT framework could be also extended with the temporal generalization logic. This approach, developed for decoding, implies training a classifier at a time point t, and testing its performance at every time points t', including t = t' (King and Dehaene, 2014). Similarly, the template tracking procedure could be performed by iterating across the localizer and main paradigm's time bins (see Figure 2, right upper inset). This allows adopting an agnostic position regarding the temporal profile of the effect of interest (e.g., Desender et al., 2019; Senoussi et al., 2020). Nonetheless, template estimation can be also carried out in a more constrained fashion. In this sense, it is possible to derive single templates, collapsing data from theoretically informed time windows, or focusing on time bins identified with data-driven approaches which seek to maximize pattern discriminability.

### 3.3. Template tracking

After activity pattern estimation, the canonical templates are tracked on the main paradigm data to assess whether and to what extent they are activated. To do so, the most promising approach incorporates the same similarity measurements within the RSA framework. Those measures have two major advantages: they have been already tested and validated in neural and simulated data (Nili et al., 2014; Walther et al., 2016), and they are remarkably flexible regarding the number of canonical templates that can be considered simultaneously.

The simplest option is to compute the Pearson correlation coefficient between the main task's activity patterns and the canonical template(s) (Wimber et al., 2015). Significantly higher correlations would be interpreted as increased activation of the targeted representation during the paradigm. Similarly, the Euclidean distance can be also used in this context, with smaller distances interpreted as a more likely re-activation of the template. In this regard, it is important to consider that correlation-based measurements are less influenced by changes in pattern scaling (for instance, when a particular template univariate signal is higher), while Euclidean distance is most robust against baseline shifts (as those entail by using separate scanning sessions across different localizer tasks). Nonetheless, further empirical work is still needed to address the performance of both metrics in the context of CTT (see Kriegeskorte and Kievit, 2013; Walther et al., 2016 to further details on the differences between correlation and Euclidean measurements in the RSA context).

Also importantly, recent research has stressed that measures based on Pearson correlation and Euclidian are highly sensitive to noise-induced bias toward more different (i.e., less correlated or more distant) activity patterns and that they also lack a meaningful zero value (Walther et al., 2016), emphasizing the need of using relative instead of absolute similarity metrics. In this regard, cross-validated versions of these distance metrics protect against this positive bias, providing more reliable estimations (Walther et al., 2016) that can be useful in the context of CTT. Among those, one popular option is the Mahalanobis distance, computed from the cross-validated Euclidean distances after performing multivariate noise normalization on the activity patterns. Nonetheless, this approach could lead to negative distance values that would be difficult to interpret as activation indexes within the CTT framework. Alternatively, cross-validated correlation distance would avoid that issue, offering less biased estimates that can be more easily interpreted. Nonetheless, it is important to stress that implementing a cross-validation procedure is less straightforward in the context of CTT than in regular single-paradigm designs. In the latter, different task runs or trials can be flexibly included in k-fold or leave-one-out cross-validation methods. In the CTT case, the main paradigm and localizer data should be treated separately. One option would consist of keeping the localizer templates fixed in the cross-validation while iterating across different and independent main task data chunks. However, future research is needed to properly characterize cross-validated distance measurements from the CTT perspective.

The above-mentioned measurements (correlation and Euclidean distance) are especially useful when the goal is to address the presence or absence of particular templates. However, they are not optimal for studies aiming to compare multiple canonical templates simultaneously, obtained either from the same or from different localizers tasks. Computing independent similarity measures for each one would not account for the variance shared across them, and as such, would not address the particular contribution of each one. Critically, other approaches as semi-partial correlations (e.g., Hebart et al., 2018; González-García et al., 2021) or multiple regression (e.g., Nastase et al., 2017; Palenciano et al., 2019) can be also incorporated during template tracking. These approaches properly partition the task variance, assessing both the unique and shared contribution of multiple templates, and as such, are highly relevant for CTT analyses.

In alternative, a classifier-based approach can be also used for template tracking, following a parallel procedure as in a cross-decoding analysis. In this case, a machine learning classifier is trained on the localizer data and tested against the task activity patterns. However, the standard implementation of this approach could restrict the flexibility of the CTT framework. Specifically, if only the classifier accuracy is considered, it will generally indicate to what extent the templates generalize to the main paradigm data instead of obtaining evidence for the individual templates' reactivation (as it happens with the above-mentioned methods). Additional output measurements can be computed to enrich the conclusions drawn with this approach. For instance, we can also extract trial-wise distances from the hyperplane, as the d values obtained with LDA classifiers (e.g., Linde-Domingo et al., 2019). These measurements are assigned to each activity pattern from the main paradigm during the classifier testing phase, and not only indicate the canonical template label assigned to the trial, but also the confidence of the classification (with higher *d* values indicating that the trial was further away from the boundary decision, and hence, more easily classified as the corresponding template label). Even more sophisticated decoding implementations have been used in the past to obtain further evidence on the reinstatement of particular canonical templates (e.g., Kok et al., 2017; Liu et al., 2019). Nonetheless, obtaining trial-wise indexes for multiple templates and comparing among them is less straightforward from the classifier-based than the RSA approach. Moreover, using decoding algorithms would impose further experimental design requirements, such as longer localizer tasks that provide enough data samples for the classifier training. Having said that, it is also important to stress that decoding classifiers can also be powerful in combination with localizers, and as such, have been successfully implemented in previous literature (e.g., Kurth-Nelson et al., 2016; Liu et al., 2019; Wimmer et al., 2020).

The selection of the measurement underlying the template tracking is one of the core decisions for a CTT experiment. Nonetheless, while previous research has documented the different methods' strengths and caveats for RSA and decoding analyses, a parallel effort is still missing in the context of using separate main task and localizer data. Hence, the impact of the metric employed as activation index (different distance measurements or classification accuracy) or the procedure followed (with or without cross-validation) is partially uncertain to date. Our recommendation is to select among the mentioned options according to the inference goals of the study. In general terms, the correlation distance, when used with a proper baseline comparison, is the simplest, more straightforward option to interpret. Either correlation distance or classification accuracy are suitable methods to address the presence of specific templates. However, when the research question requires the comparison among multiple templates, similarity measurements based on multiple regressions or semi-partial correlations can help disentangle each activity pattern's contribution. Having said that, further methodological work would be key to providing more robust recommendations in this regard.

In the scripts provided with this work, we demonstrate two implementations of template tracking on simulated MRI data (also available in the repository, simulated with The RSA Toolbox; Nili et al., 2014). First, we compute correlation-based similarity metrics, comparing two conditions' canonical templates (using one as baseline as described in Section 2.2). Second, we employ multiple regression to compare two independent localizer templates. For simplicity, the activity patterns were estimated from traditional, beta-per-run GLMs, without noise normalization, and using a ROI approach. Since the code generated was based on the Decoding Toolbox's main functions (Hebart et al., 2014), we refer the reader to its documentation to further include the different activity pattern estimation options mentioned above.

### 3.4. Statistical inference

If an RSA-based template tracking is followed, the CTT procedure will provide, for each individual participant, an activation index (either correlation or distance measure or beta weight) per canonical template, and spatial (voxels or ROI) or temporal (time bin) unit explored in the analysis. Alternatively, if a decoding-based approach is followed, for each participant and spatial or temporal unit, we will obtain either a general decoding accuracy or one or several classifier outputs associated with each activity pattern from the main paradigm considered.

To perform inference on these measurements at the group level, it is important to take into account that most of the template tracking measurements will not follow the assumptions imposed by parametric tests, and non-parametric alternatives generally are recommended. Most importantly, if the template tracking is performed using non-cross-validated similarity measurements (as standard Pearson correlation or Euclidean distance), the activation indexes will be positively biased (i.e., may be artificially inflated). In those cases, instead of addressing whether a template's activation index is above zero, the inference should be performed on the relative activation increase across templates (see Section 3.5). Finally, it is also worth noting that, as is normally the case with neuroimaging data analysis, a very large number of statistical tests will be performed, requiring the implementation of a proper multiple comparisons correction.

One possibility is to carry out Wilcoxon signed-rank tests (Wilcoxon, 1945), a non-parametrical alternative to one-sampled and paired *t-*tests employed in the past on multivariate analyses of EEG and fMRI data (Nili et al., 2014; Grootswagers et al., 2017). In this regard, paired-sample Wilcoxon tests can be used to assess relative increases in the targeted templates' activation index against the predefined baseline or control condition template. One-sample Wilcoxon test (comparing an activation index against zero) should only be used when the activation index controls for the positive bias above defined (i.e., for cross-validated measurements). A second, more comprehensive option is to perform permutation-based testing, during which the real task data labels will be shuffled in order to estimate the distribution of activation values under the null hypothesis. Interestingly, this null distribution could help in the interpretation of positively inflated distance metrics obtained during the template tracking phase. Moreover, the permutation procedures also enable the estimation of null distributions of cluster sizes (either among contiguous voxels or time points), which can be further used to control for multiple comparisons with a cluster-wise criterion (Stelzer et al., 2013), implementing either a Family-Wise-Error or a False-Discovery-Rate correction. This approach considers the spatial and temporal dependencies inherent to neuroimaging data, and enables balancing the control for false positives with a less detrimental effect on false negatives. Its implementation in multivariate analysis pipelines of fMRI and M/EEG has been popularized in recent years (Maris and Oostenveld, 2007; Stelzer et al., 2013; López-García et al., 2022).

### 3.5. Interpretation of CTT results

In general terms, CTT can be carried out to perform two broad types of inferences: either to assess the presence or absence of individual representations or to compare the activation strength of several competing templates. The former will be based on finding a systematic and specific relationship between the target canonical templates and the main paradigm's activity patterns. The latter will further require properly partitioning the main paradigms' variance to capture each template's unique contribution. In either case, to interpret the obtained activation indexes as evidence for the presence or strength of encoded information, it is important to consider the impact of contaminating effects that can bias CTT results, mainly: detecting general (instead of content-specific) effects on activity patterns and confounding the computed activation indexes with the template or localizer reliability.

Regarding the confound derived from general effects, and independently of the study's inference goal, it will be crucial to draw conclusions from relative instead of absolute template activation indexes, as it has been already stressed in different sections of this work. For that, the targeted templates should be compared against proper control or baseline templates from the same localizer task. Absolute similarity measurements can capture unspecific task variance which instead of informing about the content of brain representations, can reflect more general cognitive processes, such as unspecific perceptual processing, motor preparation or execution, or broad cognitive control or attentional sets. The nature of the similarity measurements mentioned in the tutorial (Walther et al., 2016) further emphasizes the need for relative indexes.

The second source of confounds—the reliability of the activity patterns—is especially important when several templates or localizers are compared. Finding increased similarity with a canonical template (or localizer) could be also driven by more reliable or informative, or alternatively, less noisy, activity patterns in that condition. While efficient experimental design should ensure that all the sources of canonical templates are equivalent to each other and equally similar to the main paradigm, other sources outside experimental control can influence the templates' reliability (as baseline shifts across scanning sessions, noise distribution, participant engagement, etc.). Hence, providing further empirical evidence supporting similar template reliability can help the interpretation. In this regard, previous studies (Wimber et al., 2015; González-García et al., 2021) have assessed whether alternative templates or localizers have an equivalent signal-to-noise ratio (computed using the mean *t* value linked to the similarity measurement, divided by the standard deviation), informational content (using Shannon entropy) and whether they correlate with the remaining templates from the same conditions to a similar degree (measurement identified as “correlationability”).

Finally, the interpretation would also depend on the specific comparison approach followed during the template tracking. While most of the recommendations given so far relate to RSA-based similarity measurements, machine-learning classifiers have been also widely used in previous studies combining localizers and MVPA. Thus, it is important to consider that both RSA and decoding-based measurement reflect different aspects of the addressed representational geometry. On one hand, RSA similarity measurements are more agnostic about the underlying mechanisms, and significant increases in these metrics just state that both canonical templates and the main task activity patterns are systematically closer in the estimated multidimensional representational space (Kriegeskorte et al., 2008a; Kriegeskorte and Kievit, 2013). On the other, decoding-based methods, when they provide above-chance classification accuracy, inform that the main paradigm's activity patterns could be readout by a similar linear decoder as the one interpreting the canonical templates (however, see Carlson et al., 2018; Ritchie et al., 2020). It is worth noticing, however, that the current MVPA research is still far from understanding the impact of these two analytical approaches in neural encoding, and more importantly, their cognitive implication. Having said that, it is still relevant to consider the differences between both techniques when interpreting CTT results.

## 4. Limitations and pitfalls

So far, this work has highlighted the strengths of using localizers with a CTT approach, to promote its inclusion among the MVPA tools available for cognitive neuroscientists. Nonetheless, it is also important to acknowledge a series of drawbacks that are inherent to this method and that can limit its implementation.

### 4.1. Need for appropriate localizer(s)

The main pitfall of CTT studies is related to the design of the localizer tasks. Here, we provided a set of general recommendations on experimental design, that should be operationalized under the specific research questions. However, how to achieve this goal is not always straightforward. It can be quite challenging to find localizer tasks that capture the process of interest without overlapping too much with the main paradigm or including additional, unwanted cognitive computations. Ensuring the equivalence (at the perceptual, motor, and cognitive level) among all the canonical templates and localizers included in the CTT analysis hampers, even more, the experimental design. That extrapolates to obtaining similar behavioral performance across the conditions compared to ensure equivalent participants' engagement and task difficulty. Furthermore, selecting suitable baseline conditions can be also problematic in some circumstances. Hence, even when several reliability measurements can be computed to rule-out confounds (see Section 3.5), poor experimental design decisions can seriously affect interpreting CTT results. Nonetheless, we would like to emphasize that similar design issues can also affect other MVPA techniques, due to their increased sensitivity (Hebart and Baker, 2018). Hence, acknowledging this pitfall does not invalidate the CTT approach, but instead, encourages further care and caution when designing localizers tasks for MVPA testing.

### 4.2. Additional resources

Even with elegant paradigm and localizer task designs, other CTT limitations emerge at this stage. The first one derives naturally from the use of one or several localizer task(s): they will consume additional time and participants' effort. Since both resources are particularly critical in the context of neuroimaging research, we recommend careful cost-and-benefit considerations during the study's design phase. In this regard, we encourage using CTT only when the research question of interest can be better addressed by including independent localizer blocks with more constrained task demands. Otherwise, a more parsimonious approach using single-paradigm studies and other MVPA techniques should be always preferred. In this regard, there are other decisions at the level of design (for instance, with condition-rich designs; Kriegeskorte, 2009) and analysis (for instance, using more sophisticated RSA models derived from biologically inspired deep neural networks; Kriegeskorte, 2009; Ito et al., 2022) that could be implemented to bypass the need of adding localizers. Nonetheless, and as stated in the introduction, for some research questions and cognitive domains, the inclusion of localizers (and the corresponding additional resources invested in them) may still be the most optimal strategy. In this regard, it is also important to highlight the renovated interest in psychology and cognitive neuroscience in increasing the amount of data collected from individual participants (Smith and Little, 2018). While this approach goes against the dominant paradigm, focused on achieving statistical power through greater sample sizes (Poldrack et al., 2017), it has proven to be an alternative path to detect relevant and robust effects (e.g., Poldrack et al., 2015; Newbold et al., 2020). Considering the resource requirements linked to CTT and the additional localizer(s) task(s) aligns well with this debate.

### 4.3. Considerations on the experiment's temporal structure

The CTT experimental design not only increases the overall temporal demands but also leads to further decisions on the experiment's temporal structure, which in turn may induce additional confounds. In particular, the sequence in which the main paradigm and localizers are shown can be confounded with time-on-task (reflected on both participants' fatigue and expertise), low-frequency drift artifacts, or general order effects (for instance, predictability of the transitions). Increasing the number of localizer tasks in the design will impose further difficulties in controlling these contaminating effects. In contrast, single-paradigm studies based on event-related design and trial-by-trial conditions randomization (Dale, 1999) are optimal in reducing the systematic impact of temporal confounds. Hence, it could be argued that instead of using independent localizer blocks, a preferred option would be to simply add localizer trials interspersed along the main paradigm task. This approach would still enable the comparison between the localizer and main paradigm trials' representations. However, it is important to consider that in some experimental paradigms, incorporating additional localizers' demands or rules not only will increase the overall task complexity and difficulty but can also disrupt the performance in the cognitive process of interest (Duncan et al., 2008). In consequence, separating the main paradigm and localizers' demands into independent blocks can also be required in some contexts. Thus, when addressing the viability of a CTT design, researchers should consider both the increased difficulty in controlling temporal confounds and the particularities of the cognitive process of interest.

### 4.4. Theoretical assumptions

Finally, beyond the limitations derived from experimental design, CTT also imposes additional theoretical assumptions that may hinder its applicability. Specifically, template tracking capitalizes on the generalizability of activity patterns between localizer tasks and the main paradigm. Hence, this method is highly relevant to study representations encoded in a common, abstract neural code that can transfer across cognitive contexts. This attribute is also shared by cross-decoding analysis (Kaplan et al., 2015), and far from being a limitation *per se*, generalizability has been identified as a key aspect of MVPA to broaden theories of brain function and cognition (Varoquaux and Poldrack, 2019). Having said that, it is important to acknowledge that this perspective may be difficult to reconcile with theoretical proposals based on the opposite assumption: the non-generalizability or low abstraction of neural representations. Especially regarding higher-order cognitive processes, previous evidence stresses the presence of high-dimensional, conjunctive representations that non-linearly mix multiple types of information to flexibly encode tasks goals (Rigotti et al., 2013; Kikumoto and Mayr, 2020). Such representations would be difficult to capture with CTT, since they are not expected to transfer across task contexts. Hence, CTT would not be an optimal tool to address hypotheses derived from these frameworks. Critically, other theoretical models and available evidence also support the presence of abstract, generalizable representations for higher-order cognition (Bernardi et al., 2020; Badre et al., 2021). With that, we would like to stress that CTT can be a valuable tool for studying not only sensorimotor processes but also more complex, goal-oriented cognition. However, in order to apply this technique, we must consider the nature of the hypothesized neural representations and their alignment with the theoretical assumptions imposed by CTT.

## 5. Discussion

Despite the increased interest in multivariate brain data analyses, no work to date had provided methodological guidelines on CTT. With this work, we aimed to illustrate the advantages of this technique and to discuss the crucial design and analysis decisions for its correct implementation. On one hand, we stressed the importance of carefully considering the task demands and experimental conditions in the localizer tasks(s). On the other hand, although CTT partially overlaps with (cross-) decoding and RSA, we highlighted the specific steps and decisions regarding template estimation and tracking. Moreover, we provided a comprehensive tutorial covering spatially and temporally resolved neuroimaging recordings, together with a set of scripts to implement the analysis using a popular toolbox for multivariate pattern analysis (Hebart et al., 2014). In doing so, we aimed to unify the methodology employed in previous work framed within CTT and to encourage future research in incorporating this technique as a powerful tool to investigate cognitive processes from an information-based approach.

Previous literature has evidenced the relevance of CTT, as well as related approaches combining localizers and MVPA, in addressing questions of cognitive neuroscience. The specificity of this technique, which allows targeting specific stimulus representations, has contributed to a better understanding of how cognitive processes transform the information being encoded. In the memory domain, for instance, the study by Wimber et al. (2015) showed how CTT can be informative on both excitatory and inhibitory processes underpinning adaptive forgetting. Hence, this approach could be highly relevant for exploring other competition processes that may engage both prioritization and suppression mechanisms and benefit from disentangling both influences. Similarly, CTT could be used to investigate associative processes binding multiple representations (Senoussi et al., 2016). In more general terms, CTT opens a window to explore the impact of cognitive operations in specific materials, instead of in broader categorical conditions (e.g., animate and inanimate stimuli), as is frequently the case with techniques such as decoding algorithms. We believe that tracking how task demands transform individual stimulus representations can be key to generating specific and testable hypotheses, hence contributing to the communication between theoretical frameworks and neuroimaging research.

Moreover, CTT has also provided valuable evidence for distinguishing among representational formats. For instance, in the cognitive control domain, different frameworks predict the format in which the task-relevant information (stimuli, responses, and links between them) is represented to optimally guide behavior (e.g., Hommel et al., 2001; Hommel, 2009; Rigotti et al., 2013; Badre et al., 2021). One popular perspective stresses that task content needs to be represented in an action-oriented (or procedural) code that is functionally different from encoding the same information in a more symbolic or declarative format (Brass et al., 2017). Employing CTT, González-García et al. (2021) disentangled between task representations that overlapped in content (e.g., relevant stimulus-response association) but differed in format (i.e., procedural and declarative). By using different localizers' task demands, these authors estimated procedural and declarative templates and found a greater impact of the former on both neural activity patterns and performance. Keeping constant the templates' content while manipulating their format is a key strength of CTT and extends the conclusions that can be drawn with decoding and RSA (especially in contexts where condition-rich designs cannot be incorporated). Beyond cognitive control, this methodology could also be relevant for other research contexts where theories predict specific representational formats. That is the case, for instance, of action-processing frameworks that emphasize a common encoding format across overt performance, motor planning, or even imagery (Jeannerod and Decety, 1995; Grush, 2004; Jeannerod, 2004), or embodied language proposals predicting an overlap in the representations induced by linguistic and sensorimotor processing (Barsalou, 2008). Thus, carefully designed localizer tasks could be a powerful tool to help decipher the encoding format underpinning different cognitive processes. Moreover, the characterization of the representational format can be further enriched by combining CTT with other analytical approaches targeting this encoding dimension. For example, in a recent study, Kwak and Curtis (2022) employed retinotopic mapping and image reconstruction techniques to characterize the format of abstract feature representations held in working memory. To do so, reconstructed retinotopic representations were compared against activity patterns found during the working memory retention interval, following a similar logic as in CTT. This novel approach opens a window to further expand the CTT framework, including not only estimated but also reconstructed canonical templates.

Furthermore, CTT is particularly relevant for the exponentially growing literature aiming to characterize the information coded in unconstrained or spontaneous brain activity patterns (Matusz et al., 2019; Sonkusare et al., 2019; Liu et al., 2022). Most of the neuroimaging research on cognitive neuroscience relies on the experimental control provided by carefully designed paradigms. Nonetheless, recent work has stressed the limitations of using restricted task and stimulus spaces to explore brain functions, which evolved to deal with complex, multidimensional information (Nastase et al., 2020). That has motivated the employment of naturalistic experimental setups (Sonkusare et al., 2019), which include movie visualizations (Hasson et al., 2004), natural speech perception (Huth et al., 2016; Hamilton and Huth, 2018), free recall tasks (Chen et al., 2016), etc. Simultaneously, another promising path has entailed the analysis of the content represented in spontaneous activity during resting state periods (Liu et al., 2019; Kim et al., 2020). While these approaches provide unprecedented ecological validity, they do so at the cost of making the implementation of common multivariate analyses more challenging. Localizers have proved to be highly useful in this context since they enable isolating the target representations which are later used to identify the content during unconstrained or resting brain activity (e.g., Kurth-Nelson et al., 2016; Liu et al., 2019; Wimmer et al., 2020). Nonetheless, most of this research has been developed from the decoding perspective, training classifiers with the localizer data and testing them against spontaneous activity patterns. With our work, we aim to expand the methodological tools available for that purpose, providing a flexible framework where different metrics can adapt to the nature of the localizer and experimental setup.

Beyond its potentiality, it is also important to acknowledge that the lack of previous methodological publications on CTT calls for further research. For instance, regarding experimental design, previous literature has discussed the decisions that optimize multivariate pattern analyses, with different recommendations being made for studies employing decoding (Mur et al., 2009) and RSA (Kriegeskorte et al., 2008a). Nonetheless, the design of localizer tasks from the multivariate or information-based framework had not been addressed so far. That contrasts with the considerable attention that this issue received from the activation-based perspective (Friston et al., 2006; Saxe et al., 2006). The debate regarding functional, univariate localizers has encompassed both the abstract theoretical implications (Friston et al., 2006) and concrete paradigms and stimuli employed (e.g., Fox et al., 2009; Berman et al., 2010). While the localizer logic did extrapolate to the multivariate framework, a parallel discussion has not taken place yet in this domain. As a result, similar tasks used in the past to functionally define regions of interest are now being used to isolate the underlying neural representations. In our work, we aimed to explicitly state different issues concerning the selection of the localizer(s) task demands and experimental conditions, as well as their relationship with the inference goals of the study. Nonetheless, we are aware that additional work is required to refine this technique. We consider that a key future direction could entail the elaboration of a taxonomy of localizer tasks that can be employed to evoke different representational formats and contents. Such a strategy would facilitate the experimental design of CTT experiments, maximize the reproducibility of the results, and facilitate collaborative data sharing.

Similarly, the analysis procedure *per se* could be further optimized. In this work, we aimed to synthesize the different approaches previously used in studies akin to CTT and to extend the methodology from decoding and RSA which could be compatible with this technique. In doing so, we emphasized two steps central to CTT: the estimation of the canonical templates and the tracking procedure. The former capitalizes on the robustness and stability of the extracted neural representations. Inspired by previous work, we recommended employing traditional run-wise GLM estimation to extract the activity patterns from fMRI data and trial-averaging in the case of M/EEG. For the latter step, template tracking, we suggested the computation of similarity measurements (as correlation-based similarity or multiple regression beta weights), derived from RSA, to maximize the flexibility during pattern comparison. We believe that our proposed step-by-step procedure fills a gap in the literature and would be useful to guide future research. Nonetheless, we also highlight that future empirical work will be key in adding more support to the template estimation and tracking process here described. For instance, while the similarity measurements included had been validated within the context of RSA, they are frequently applied to similarity structures instead of activity patterns (for instance, multiple regression beta weights). Future work using real and simulated data will provide a more exhaustive validation of these measurements in the context of localizer-driven templates.

## 6. Conclusion

The introduction of multivariate analyses in cognitive neuroscience has opened a window to a more mechanistic, representational understanding of brain function. Aiming to extend the available methods, here we described a third MVPA implementation, CTT, which empirically estimates specific neural representations from localizer tasks, and assesses its (re)activation during independent cognitive paradigms. While CTT is tightly related to decoding and RSA, we highlighted how it can complement these techniques thanks to its specificity and its ability to tap into the representational format. To facilitate its implementation in future studies, we provided a detailed tutorial regarding experimental design and data analyses and identified the main limitations and pitfalls linked to this technique. With these guidelines, we aimed to extend the analysis to both spatially and temporally-resolved datasets. To further facilitate CTT implementation, we also provided a series of scripts to carry out the analysis on MRI data. Although future work will be key to further improving CTT, we believe its inclusion among the multivariate tools employed in neuroimaging research will significantly contribute to addressing cognitive phenomena.

## Author contributions

AP, MS, SF, and CG-G contributed to the conceptualization and methodology of the current work. AP wrote the first version of the manuscript and developed the scripts provided. All authors contributed to manuscript revision, read, and approved the submitted version.
